# Investigation of bioluminescence-based assays for determination of kinetic parameters for the bifunctional *Neisseria meningitidis* serogroup W capsule polymerase

**DOI:** 10.1186/s13104-021-05831-1

**Published:** 2021-11-18

**Authors:** Laleh Sheikhi Moghaddam, Ayobami Adegbite, Pumtiwitt C. McCarthy

**Affiliations:** 1 Bioenvironmental Sciences Program, Morgan State University, 1700 East Cold Spring Lane, Baltimore, MD 21251 USA; 2 Department of Chemistry, Morgan State University, 1700 East Cold Spring Lane, Baltimore, MD 21251 USA

**Keywords:** Bioluminescence assay, CMP-Glo, UDP-Glo, Kinetics, *Neisseria meningitidis*

## Abstract

**Objective:**

*Neisseria meningitidis* is a Gram-negative bacterium that causes meningitis. *N. meningitidis* serogroup W (*Nm*W) capsule polymerase synthesizes capsular polysaccharide of this serogroup. This enzyme could be a tool for meningococcal glycoconjugate vaccine development. Our long-term goal is to control activity of the *Nm*W capsule polymerase for production of defined carbohydrates for vaccines. The enzyme lacks a simple, high-throughput activity assay. Here, we describe the use of high-throughput bioluminescence assays (CMP-Glo and UDP-Glo by Promega) to investigate *Nm*W capsule polymerase activity. These assays detect free nucleotides produced during transfer of sugar from UDP-Galactose and CMP-Sialic Acid to an acceptor. Kinetic studies using *Nm*W hydrolyzed polysaccharide (PS) acceptor are described as well as preliminary work with a sialic acid trimer (DP3) acceptor.

**Results:**

In CMP-Glo kinetic studies, with constant donor (80 µM) and varied *Nm*W hydrolyzed polysaccharide (0–2000 µg/mL), a K_m_ of 629.2 ± 101.4 µg/mL and a V_max_ of 0.8965 ± 0.05823 µM/min was obtained. Using UDP-Glo, K_m_ and V_max_ values of 13.84 ± 9.675 µM and 0.6205 ± 0.1331 µM/min were obtained with varied CMP-NeuNAc (0–80 µM) and constant acceptor (400 µg/mL) and UDP-Gal (80 µM). This is the first report of using bioluminescence assays for *Nm*W kinetics.

**Supplementary Information:**

The online version contains supplementary material available at 10.1186/s13104-021-05831-1.

## Introduction

*Neisseria meningitidis* is a gram-negative bacterium that causes most cases of bacterial meningitis [1]. Of the 13 serogroups of the bacteria, there are six to which disease is attributed [1–3]. Each serogroup is defined by its capsular polysaccharides. The capsule polymerase enzymes, responsible for biosynthesis of capsular polysaccharides, from the six pathogenic serogroups (A, B, C, W, Y and X) have been characterized to varying degrees [4–15]. Our current focus is the *Neisseria meningitidis* serogroup W (*Nm*W) capsule polymerase enzyme. This bifunctional enzyme transfers sialic acid and galactose from two nucleotide donor substrates (CMP-Neu5Ac and UDP-Gal) to an acceptor during synthesis of capsular polysaccharide. The long-term goal is to gain insight into the *Nm*W capsule polymerase as a tool for controlled synthesis of carbohydrates for use in glycoconjugate vaccines [16]. Glycoconjugate vaccines with defined carbohydrate length and well-characterized attachment to carrier proteins will be key to maximizing vaccine efficacy [17].

There are no assays described in the current *Nm*W capsule polymerase literature that allow for determination of the kinetic parameters of the enzyme in a simple high-throughput manner using unlabeled acceptors. At the time we began this work in 2018, the only report of kinetics was our previous work which used a continuous, absorbance-based assay with unlabeled acceptors [18]. In 2020, the Chen lab published elegant work using one-pot multienzyme synthesis (OPME) of chromophore-labelled oligosaccharides to determine kinetics in an HPLC-based assay [8]. In this work, we investigate commercially available bioluminescence-based assays (CMP-Glo and UDP-Glo) as tools to determine kinetics of the *Nm*W capsule polymerase. We selected these kits for their high-throughput 96-well format, sensitivity and the ability to use unlabeled acceptors. These kits detect free UDP and CMP produced by glycosyl transfer [19–21] (Additional file 1: Fig. S1). UDP-Glo or CMP-Glo nucleotide detection reagents (NDR, each containing a proprietary luciferase) are added to sample on the 96 well plates to quench the glycosyltransferase reaction. Any free CMP or UDP present is converted to ATP and luminescence is produced and monitored. Thus, increased luminescence correlates with increased ATP which correlates with more glycosyltransferase activity. Here we describe our initial efforts to obtain kinetics using both kits and lessons learned from adapting these assays for the bifunctional *Nm*W capsule polymerase.

## Main text

### Material and methods

#### Growth, expression, and purification of the Neisseria meningitidis serogroup W capsule polymerase

The enzyme was recombinantly overexpressed in *E. coli* KRX cells, purified and characterized according to a published procedure [18].

#### CMP-Glo bioluminescence assays

To determine optimal amounts of enzyme to use, assays were performed with different amounts of purified serogroup W enzyme (0 as a control, 50, 125, 250, 500, 750, 1000, and 1250 ng for reaction), CMP-NeuNAc (80 µM), UDP-Gal (80 µM), DTT (100 µM each), hydrolyzed serogroup W Acceptor (400 µg/mL), in a buffer (50 mM Tris, 20 mM MgCl_2_, 2 mM DTT, pH 8.0) established in the literature [4, 5, 18]. Reactions were run for 1 h at room temperature (RT) in 96-well plates with shaking to mix and quenched by adding CMP-Glo NDR. Luminescence was monitored on a Promega GloMax Navigator. All other CMP-Glo experiments were performed as described except with these modifications: to determine the optimal nucleotide donor concentrations—50 ng of enzyme was used, CMP-NeuNAc and UDP-Gal were varied (0–100 µM); To determine activity effects of removing one component—either NmW enzyme, acceptor, CMP-NeuNAc or UDP-Gal was absent; Time quench experiments—NmW enzyme (50 ng) was absent or present and quenched after 1, 5, 10,15, and 20 min; Kinetic studies—either acceptor was varied (0–2000 µg/mL acceptor) or CMP-NeuNAc (0–80 µM); reactions were quenched after 10 min. For all bioluminescence studies, GraphPad Prism 8.0 software was used to graph and analyze results.

#### UDP-Glo bioluminescence assays

All studies performed with the UDP-Glo kit and reagents were done in a similar manner as described for CMP-Glo except as described. Specific modifications were the use of 1250 ng of *Nm*W capsule polymerase in experiments to determine activity effects of removing one component and in time quenching experiments. In reactions using DP3 as an acceptor, 2 mM was used except in studies to determine optimal acceptor concentration (0–4 mM DP3 acceptor was used).

#### NmW capsule polymerase elongation of DMB-labelled hydrolyzed W polysaccharide

Hydrolyzed W capsular polysaccharide (10 mg/mL) was prepared as described previously [18]. The hydrolyzed NmW capsular polysaccharide was labeled with DMB according to a published procedure [22] to give a final concentration of 5 mg/mL hydrolyzed sugar. Activity was tested by reaction with NmW capsule polymerase with DMB-labelled hydrolyzed W polysaccharide (0.25 mg/mL), DTT (2 mM) in the presence or absence of 2 mM CMP-NeuNAc and 2 mM UDP-Gal respectively in the same buffer used in other studies. Control reactions contained no enzyme. Fluorescence detection was checked by HPLC using previously described conditions [7, 23] after 15 h incubation at 37 ºC. GraphPad Prism 8.0 was used to graph chromatogram results.

## Results and discussion

The long-term goal of this research is to control activity of the *Nm*W capsule polymerase for production of well-defined carbohydrates for glycoconjugate vaccines. This work describes the application of facile, high-throughput assay methods to advance this goal. Results described here are the culmination of 55 individual experiments in which two or three replicates were performed.

### Differences in reactivity observed between UDP-Glo and CMP-Glo assays

In efforts to determine the optimal conditions to perform the enzyme reactions using these kits, a series of reactions (containing CMP-NeuNAc, UDP-Gal, DTT, hydrolyzed serogroup W polysaccharide acceptor, with or without enzyme) were performed in which the only component varied was the amount of enzyme. The results for the UDP-Glo assay (Additional file 2: Fig. S2A) show increase in activity as the amount of enzyme increases (max. with 1000 ng). The results for the CMP-Glo assay (Additional file 2: Fig. S2B), a measure of sialyltransferase activity, indicate a bell-shaped activity curve (max. with 50 ng). This was an unexpected result as it was assumed that the same enzyme concentration would be used for both assay kits. However, these results suggested that the activities of the two catalytic domains were not tightly correlated under these conditions. Nevertheless, the enzyme amount selected for further studies using the CMP-Glo assay was 50 ng. vs. 750 ng of enzyme to be used in the UDP-Glo assay because the luminescence output was comparable.

### CMP-Glo kinetic assays using hydrolyzed serogroup W acceptor

**Fig. 1:**
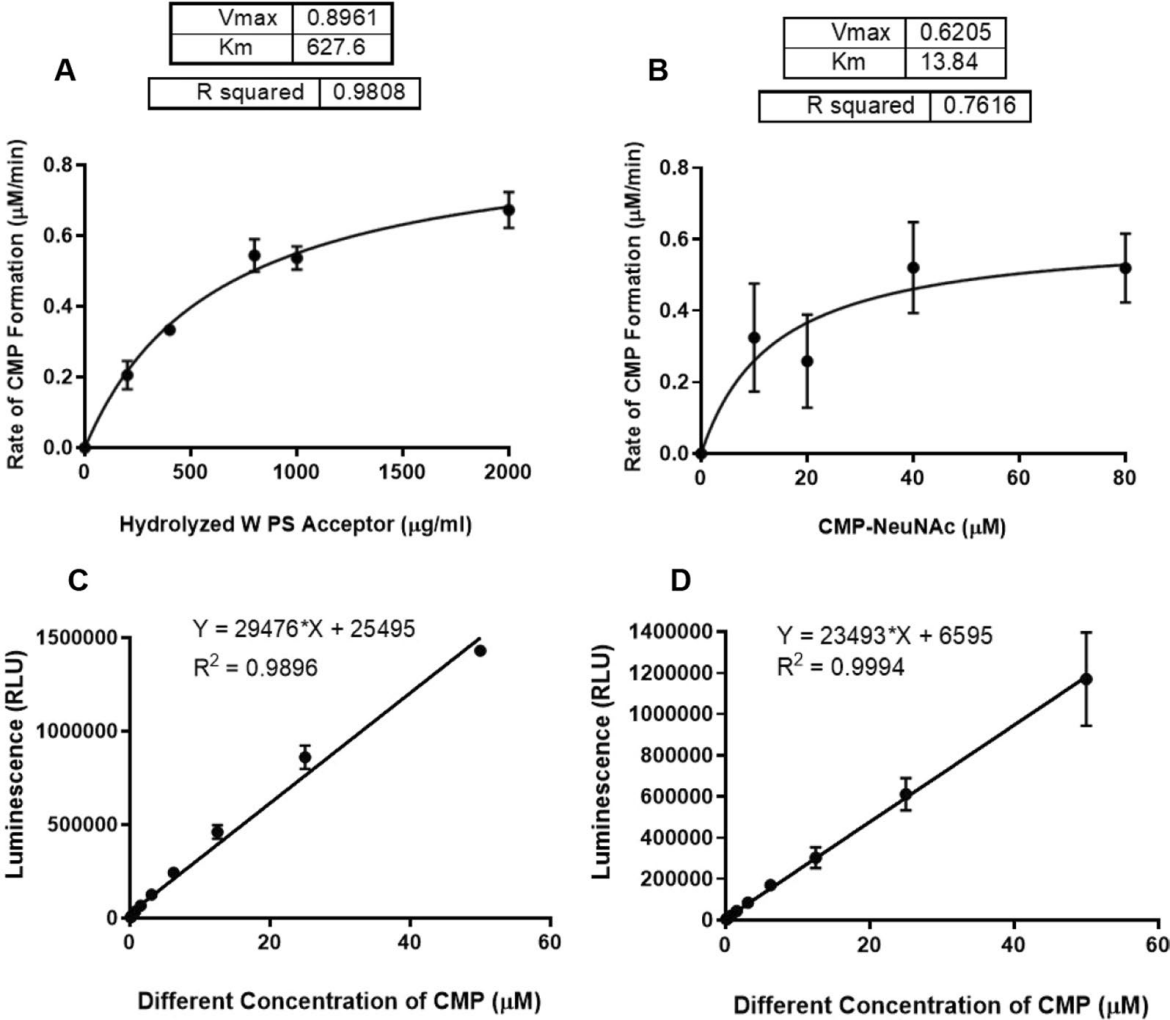
Michaelis Menten plots of kinetics data obtained using CMP-Glo with hydrolyzed serogroup W acceptor and corresponding standard curves. **A** Data with varying acceptor (0–2000 µg/mL) and other components held constant. K_m_ and V_max_ determined to be 629.2 ± 101.4 µg/mL and a V_max_ of 0.8965 ± 0.05823 µM/min. **B** Data with varying CMP-NeuNAc (0–80 µM) and other components held constant. K_m_ and V_max_ were determined to be 13.84 ± 9.675 µM and 0.6205 ± 0.1331 µM/min. **C** Standard Curve for panel **A**. **D** Standard Curve for panel **B**. (These are one representative example of three individual experiments. All experiments were run with two replicates. Data point indicates the mean and error bars represent standard deviation)

With knowledge of how much serogroup W enzyme to use, both the optimal amount of nucleotide donors and the linearity of the enzyme reaction were investigated. The optimal amount of luminescence was obtained with 80 µM CMP-NeuNAc and UDP-Gal in both kits. In addition, the enzymatic reactions were found to be linear over 10 min (Additional file 3: Fig. S3). Initially, kinetic measurements used only the CMP-Glo assay. For all kinetic assays, one component (either UDP-Gal, CMP-NeuNAc or hydrolyzed acceptor) was varied while all other components were held constant. When both nucleotide donor sugars were constant (at 80 µM each) and the amount of hydrolyzed serogroup W acceptor was varied (0–2000 µg/mL), a K_m_ value of 629.2 ± 101.4 µg/mL and a V_max_ of 0.8965 ± 0.05823 µM/min (Fig. 1A) were obtained. In kinetic studies with varied CMP-NeuNAc (0–80 µM) and constant acceptor, a K_m_ and V_max_ values were obtained 13.84 ± 9.675 µM and 0.6205 ± 0.1331 µM/min (Fig. 1B). This data set showed more variability as evidenced by the error bars and reduced R^2^ value. The related standard curves show the data is reliable (R^2^  > 0.95) (Fig. 1C, D).

### Hydrolyzed serogroup W polysaccharide acceptor contains mostly sialylated material

**Fig. 2:**
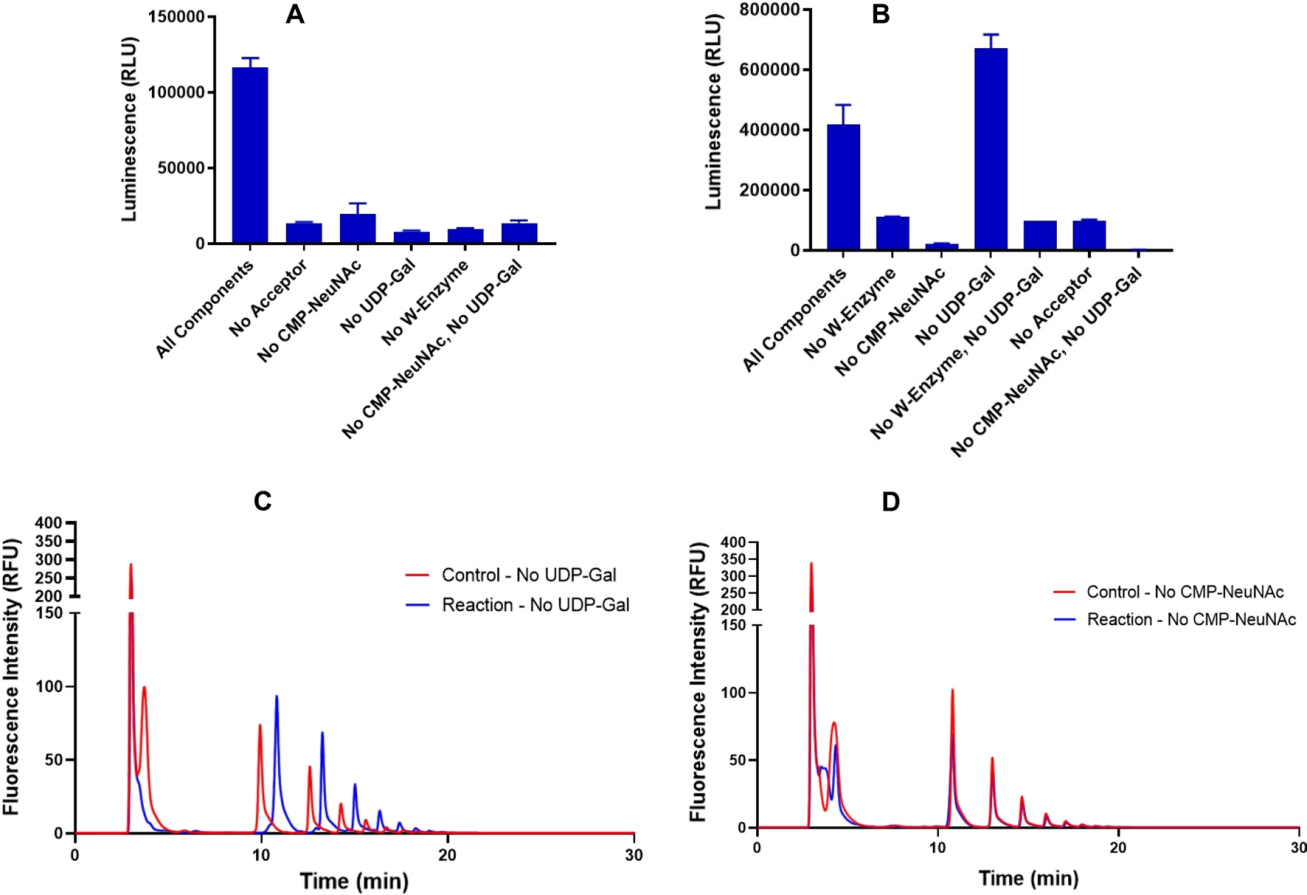
*Nm*W capsule polymerase activity in the absence of selected substrates. **A** UDP-Glo assay: maximum activity observed in presence of all components. **B** CMP-Glo assay: maximum activity observed in the absence of UDP-galactose. Panels **A** and **B** indicate representative examples of three individual experiments. All experiments were run with two replicates. Top of bar graph indicates the mean and error bars represent standard deviation. **C** HPLC chromatogram of *Nm*W capsule polymerase elongation of hydrolyzed polysaccharide acceptor in the absence of UDP-galactose and **D** in the absence of result shows control and reaction with no CMP-NeuNAc. Panel **C** shows more peaks with later retention time compared with control indicating sialylation. Panel **D** indicates no retention time difference between control and reaction except for one new peak evolved at an earlier retention time indicating galactosylation. RLU (Relative Light Unit) represents the amount of light produced by luminescence and RFU is (Relative Fluorescence Unit) for fluorescence

Because of the continued variability in the data, further confirmation that the change in luminescence observed was enzyme-mediated was needed. A series of reactions in the absence of selected components was performed using both bioluminescence kits. Galactosyltransferase activity (as observed using UDP-Glo) was seen only in the presence of all components as expected (Fig. 2A). The results of monitoring sialyltransferase activity (using CMP-Glo) were unexpected. There was an enzyme-mediated increase in activity in the absence of UDP-Gal (Fig. 2B). To gain more understanding into this finding, the products of enzymatic elongation of DMB-labeled hydrolyzed acceptor by the serogroup W capsule polymerase was visualized by anion exchange HPLC-FD. The goal was to observe whether there was any change in the chromatogram in the presence of the capsule polymerase, acceptor and with no UDP-Gal present or with no CMP-NeuNAc. As reported previously by Romanow et al., there are signature peak retention time shifts observed in elongated fluorescent products [4]. Decreased retention time indicates addition of galactose (due to the decrease in polarity by addition of the neutral sugar) and increased retention time indicates addition of sialic acid. Our data suggests that the hydrolyzed acceptor being used was primarily galactosylated (Fig. 2C, D). In the absence of CMP-NeuNAc and the presence of UDP-Gal, there is only a shift of one peak, and this is towards decreased retention time. In contrast, when CMP-NeuNAc is included and UDP-Gal omitted, there is a shift of nearly all remaining peaks towards increased retention time suggesting sialylation. At this point, it was unclear whether there was preferential hydrolysis of the polysaccharide or whether there was preferential labeling during incubation with DMB [22]. The DMB dye will only label free reducing end sialic acids so this phenomenon may influence the products that are visualized. We transitioned to a well-defined oligosaccharide which is a known substrate of the enzyme: a trimer of α, 2–8 linked sialic acid [4, 5].

#### CMP-Glo assay optimization with sialic acid trimer

While the UDP-Glo kit includes commercially available ultrapure UDP-Gal (essential in avoiding high background rates) there is no commercially available ultrapure CMP-NeuNAc. Our source of CMP-NeuNAc was the highest purity commercially available, [guaranteed 97% by Nacalai-Tesque and verified by HPLC analysis (not shown)] yet this seemingly small 3% impurity was having a large effect on results because of the sensitivity of the assay. To circumvent this, CMP-NeuNAc solutions were pre-treated with alkaline phosphatase (AP) (Additional file 4: Fig. S4A, Additional file 5). This enzyme removes phosphoryl groups from nucleotide mono- and diphosphates [24]. There were decreased levels of background CMP after this pre-treatment. DP3 trimer was also subjected to AP treatment with no change observed (Additional file 4: Fig. S4B). Despite this, there was still a considerable amount of unexplainable background luminescence (results not shown). The decision was made to focus solely on the UDP-Glo assay for subsequent studies with DP3 acceptor and continue AP pre-treatment of CMP-NeuNAc because better luminescence was observed (Additional file 6: Fig. S5A, B).

#### UDP-Glo assay optimization with sialic acid trimer

**Fig. 3:**
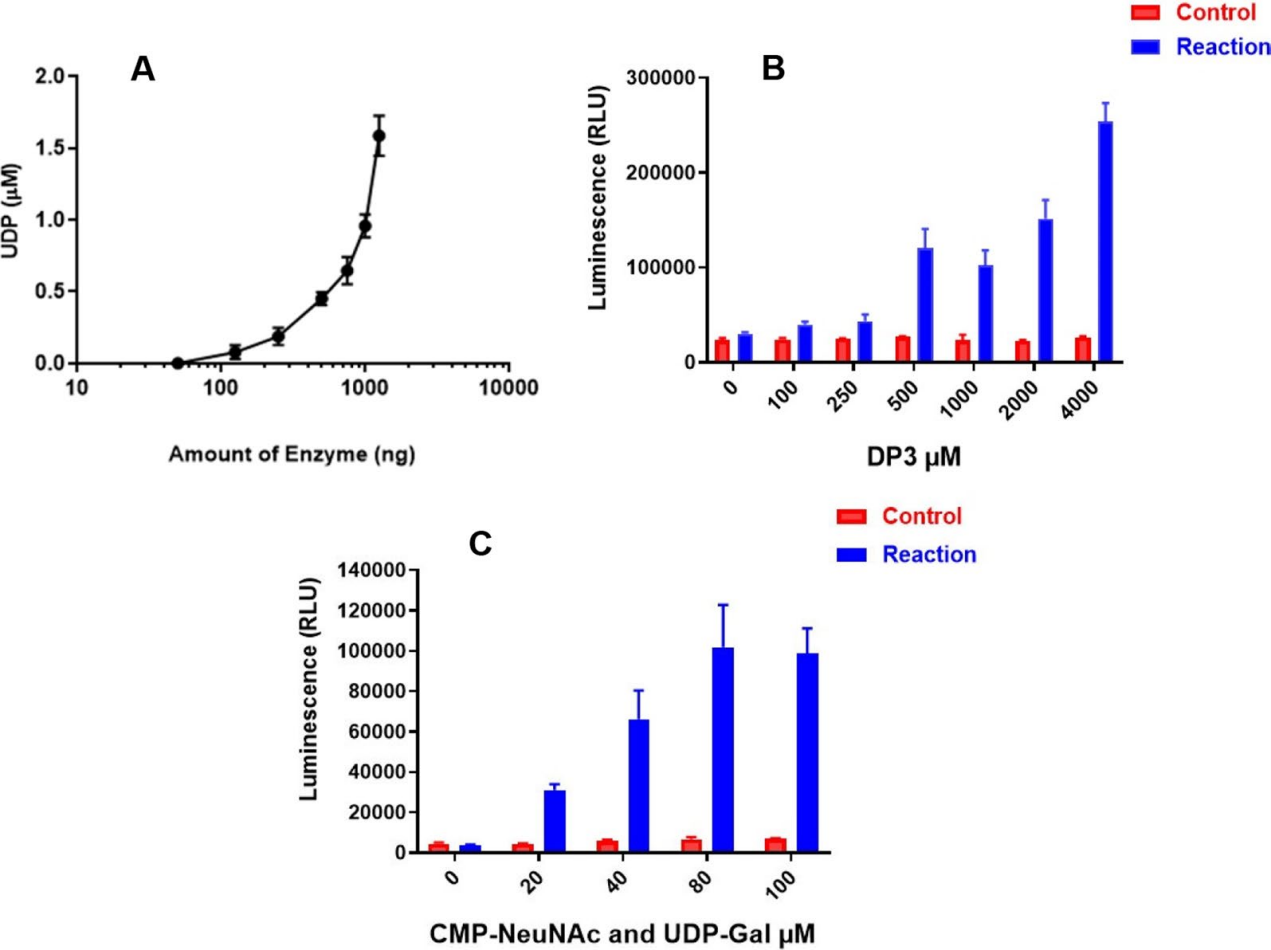
UDP-Glo assay optimization with sialic acid trimer (DP3). **A** Assay results with different *Nm*W enzyme amounts with maximum activity observed with 1250 ng enzyme. **B** Assay results using different concentrations of DP3 acceptor indicate maximum activity with 4 mM DP3. **C** Results with different concentrations of CMP-NeuNAc and UDP-Gal show more activity in the presence of 80 µM of both donor sugars (Mean  = 1,01,833 RLU) vs 100 µM (98,566.7). For panel **A**, the data point indicates the mean and error bars represent standard deviation. For panels **B** and **C**, top of bar graph indicates the mean and error bars represent standard deviation. Each panel illustrates representative examples of three individual experiments. All experiments were run with three replicates

Similar optimization assays were performed with sialic acid trimer using the UDP-Glo system. The trends mirrored those observed with hydrolyzed acceptor. Namely, there was an increase in activity with increasing levels of enzyme present in the reaction (Fig. 3A). The highest signal was seen with 4 mM DP3 as an acceptor and there was very little background observed in the control reactions (Fig. 3B). Results for the optimum amount of nucleotide donor sugar to use and the time course of the reaction were like our previous observations with hydrolyzed serogroup W sugar. The optimal luminescence was obtained with 80 µM CMP-NeuNAc and UDP-Gal (Fig. 3C) and the enzymatic reaction was found to be linear over 10 min (Additional file 7: Fig. S6).

## Conclusions

The work described here represents the first literature report of kinetics of the *Nm*W capsule polymerase using non-chromophore labelled acceptors and commercially available bioluminescence kits. Although *Nm*W hydrolyzed capsular polysaccharide was not the ideal acceptor for continuation of this work, the lessons learned from these studies were essential and continue to inform our studies using well-defined acceptors. Future studies will focus on mutational approaches to control carbohydrate synthesis by the *Nm*W capsule polymerase to develop well-defined glycoconjugate vaccines.

## Limitations

The original acceptor used, *Nm*W hydrolyzed sugar, was not a suitable acceptor to move forward with due to not being well-defined and being mostly sialylated material. When using a well-defined acceptor in the CMP-Glo assay, high background signals in control reactions were observed that were not remedied by AP treatment. Current efforts are focused on solely using the UDP-Glo assay for kinetic studies with the *Nm*W capsule polymerase and DP3-based acceptors.

## Supplementary Information

**Additional file 1: Figure S1.** A schematic of the relationship between free nucleotides produced by the NmW capsule polymerase reaction and their use in the UDP-Glo and CMP-Glo reactions. Modified from Sharyan et al. [18].

**Additional file 2: Figure S2.** Bioluminescence assay with different amount of W-Enzyme and hydrolyzed sugar. **A** With UDP-Glo assay increasing enzyme amount is correlated to increasing activity (maximum with 1000 ng). **B** In CMP-Glo assay maximum activity was observed with 50 ng of enzyme. Data point indicates the mean and error bars represent standard deviation. Each panel illustrates representative examples of three individual experiments. All experiments were run with two replicates.

**Additional file 3: Figure S3.** Time course reactions using hydrolyzed sugar acceptor. **A **CMP-Glo results: The plot of results linearity over 10 minutes whereas for **B** UDP-Glo results: this linearity was obtained over 15 minutes. All reactions run in duplicate. Data point indicates the mean and error bars represent standard deviation. Each panel illustrates representative examples of four individual experiments. All experiments were run with two replicates.

**Additional file 4: Figure S4.** Alkaline phosphatase treatment of *Nm*W substrates. **A **CMP-NeuNAc solutions were pre-treated with alkaline phosphatase. **B **DP3 trimer was subjected to phosphatase treatment. Each panel illustrates representative examples of three individual experiments. All experiments were run with three replicates.

**Additional file 5:** Supplemental Methods.

**Additional file 6: Figure S5.** *Nm*W capsule polymerase reaction using **A** untreated CMP-NeuNAc and **B **AP-treated CMP-NeuNAc in the UDP-Glo assay. The background signal with untreated CMP-NeuNAc was higher compared to treated. Higher overall luminescence signal was observed with treatment. Each panel illustrates representative examples of three individual experiments. All experiments were run with three replicates.

**Additional file 7: Figure S6.** Time course experiment using DP3 acceptor in the UDP-Glo assay. The enzymatic reaction was found to be linear over 10 minutes. Data point indicates the mean and error bars represent standard deviation. This panel illustrates representative examples of three individual experiments. All experiments were run with three replicates.

## Data Availability

The datasets used and/or analyzed during the current study are available from the corresponding author on reasonable request.

## Abbreviations

AP: Alkaline phosphatase
CMP-Glo assay: CMP Glycosyltransferase assay
CMP-NeuNAc: Cytidine monophosphate sialic acid
DMB: 1,2-Diamino-4,5-methylenedioxybenzene
DTT: Dithiothreitol
K_m_: Michaelis constant
NDR: Nucleotide detection reagent
NmW: *Neisseria meningitidis* serogroup W
RLU: Relative light unit
UDP-Gal: Uridine diphosphate galactose
UDP-Glo assay: UDP glycosyltransferase assay
V_max_: Maximal velocity

## References

[1] Borrow R, Alarcón P, Carlos J, Caugant DA, Christensen H, Debbag R (2017). The Global Meningococcal Initiative: global epidemiology, the impact of vaccines on meningococcal disease and the importance of herd protection. Expert Rev Vaccines.

[2] Stephens DS (2007). Conquering the meningococcus. FEMS Microbiol Rev.

[3] Tzeng YL, Thomas J, Stephens DS (2016). Regulation of capsule in *Neisseria**meningitidis*. Crit Rev Microbiol.

[4] Romanow A, Keys TG, Stummeyer K, Freiberger F, Henrissat B, Gerardy-Schahn R (2014). Dissection of hexosyl- and sialyltransferase domains in the bifunctional capsule polymerases from *Neisseria**meningitidis* W and Y defines a new sialyltransferase family. J Biol Chem.

[5] Romanow A, Haselhorst T, Stummeyer K, Claus H, Bethe A, Mühlenhoff M (2013). Biochemical and biophysical characterization of the sialyl-/hexosyltransferase synthesizing the meningococcal serogroup W135 heteropolysaccharide capsule. J Biol Chem.

[6] Mosley SL, Rancy PC, Peterson DC, Vionnet J, Saksena R, Vann WF (2010). Chemoenzymatic synthesis of conjugatable oligosialic acids. Biocatal Biotransform.

[7] McCarthy PC, Saksena R, Peterson DC, Lee CH, An Y, Cipollo JF (2013). Chemoenzymatic synthesis of immunogenic meningococcal group C polysialic acid-tetanus Hc fragment glycoconjugates. Glycoconj J.

[8] Li R, Yu H, Muthana SM, Freedberg DI, Chen X (2020). Size-controlled chemoenzymatic synthesis of homogeneous oligosaccharides of *Neisseria**meningitidis* W capsular polysaccharide. ACS Catal.

[9] Muindi KM, McCarthy PC, Wang T, Vionnet J, Battistel M, Jankowska E (2014). Characterization of the meningococcal serogroup X capsule N-acetylglucosamine-1-phosphotransferase. Glycobiology.

[10] Ming SA, Cottman-Thomas E, Black NC, Chen Y, Veeramachineni V, Peterson DC (2018). Interaction of *Neisseria**meningitidis* Group X N-acetylglucosamine-1-phosphotransferase with its donor substrate. Glycobiology.

[11] Ming SA, Caro NC, Lanz N, Vionnet J, Vann WF (2019). Effect of acceptor chain length and hydrophobicity on polymerization kinetics of the *Neisseria**meningitidis* group C polysialyltransferase. Biochemistry.

[12] Freiberger F, Claus H, Günzel A, Oltmann-Norden I, Vionnet J, Mühlenhoff M (2007). Biochemical characterization of a *Neisseria**meningitidis* polysialyltransferase reveals novel functional motifs in bacterial sialyltransferases. Mol Microbiol.

[13] Fiebig T, Freiberger F, Pinto V, Romano MR, Black A, Litschko C (2014). Molecular cloning and functional characterization of components of the capsule biosynthesis complex of *Neisseria**meningitidis* serogroup A: toward in vitro vaccine production. J Biol Chem.

[14] Fiebig T, Berti F, Freiberger F, Pinto V, Claus H, Romano MR (2014). Functional expression of the capsule polymerase of *Neisseria**meningitidis* serogroup X: a new perspective for vaccine development. Glycobiology.

[15] Böhm R, Freiberger F, Stummeyer K, Gerardy-Schahn R, von Itzstein M, Haselhorst T (2010). *Neisseria**meningitidis* serogroup B polysialyltransferase: insights into substrate binding. ChemBioChem.

[16] McCarthy PC, Sharyan A, Sheikhi Moghaddam L (2018). Meningococcal vaccines: current status and emerging strategies. Vaccines.

[17] Adegbite A, McCarthy PC (2021). Recent and future advances in the chemoenzymatic synthesis of homogeneous glycans for bacterial glycoconjugate vaccine development. Vaccines.

[18] Sharyan A, Gonzalez C, Ukaegbu O, Powell K, McCarthy PC (2018). Determination of the binding affinities of *Neisseria**meningitidis* serogroup W capsule polymerase with two nucleotide sugar substrates. BMC Res Notes.

[19] Promega. UDP Glo bioluminescence assay. Glycosyltransferase assays technical manual. https://www.promega.com/resources/protocols/technical-manuals/101/udp-glo-glycosyltransferase-assay-protocol/. Accessed 8 May 2021.

[20] Promega. UMP/CMP Glo bioluminescence assay. Glycosyltransferase assays technical manual. https://www.promega.com/resources/protocols/technical-manuals/500/ump-cmpglo-glycosyltransferase-assay-protocol/. Accessed 8 May 2021.

[21] Das D, Walvoort MT, Lukose V, Imperiali B (2016). A rapid and efficient luminescence-based method for assaying phosphoglycosyltransferase enzymes. Sci Rep.

[22] Lin SL, Inoue S, Inoue Y (2000). Acid-base properties of the reaction product of sialic acid with fluorogenic reagent, 1,2-diamino-4,5-methylenedioxybenzene (DMB). Carbohyd Res.

[23] Peterson DC, Arakere G, Vionnet J, McCarthy PC, Vann WF (2011). Characterization and acceptor preference of a soluble meningococcal group C polysialyltransferase. J Bacteriol.

[24] Osman Sheikh M, Wells L. Preparation of low background sugar-nucleotide donors for use in the UDP-Glo™ glycosyltransferase assay. https://www.promega.com/Resources/PubHub/Preparing%20Low%20Background%20Sugar%20Nucleotide%20Donors%20for%20UDPGlo%20Glycosyltransferase%20Assay%20Article/?fq=osman. Accessed 8 May 2021.

