# Supplementary figures and images for: Investigation of bioluminescence-based assays for determination of kinetic parameters for the bifunctional Neisseria meningitidis serogroup W capsule polymerase
Source: BMC Res Notes. 2021 Nov 18;14:417. doi: 10.1186/s13104-021-05831-1 (PMC8600345; doi:10.1186/s13104-021-05831-1)

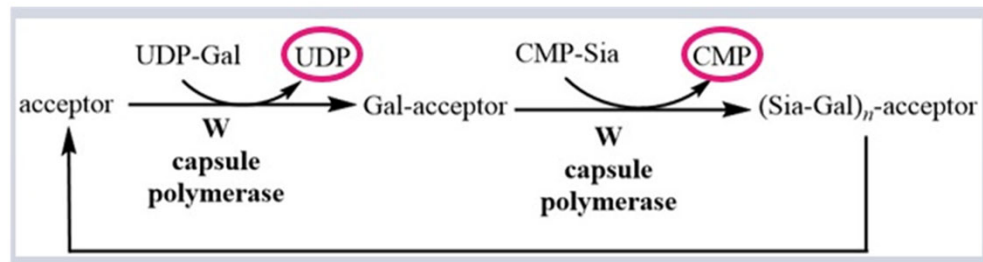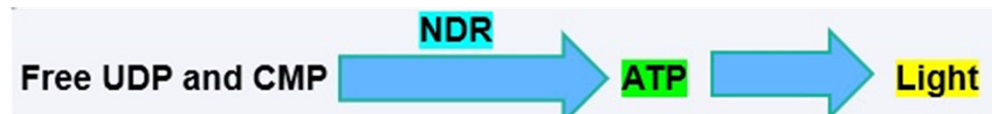

Supplement: Supplementary file 1 — Additional file 1: Figure S1. A schematic of the relationship between free nucleotides produced by the NmW capsule polymerase reaction and their use in the UDP-Glo and CMP-Glo reactions. Modified from Sharyan et al. [18]. [file 13104_2021_5831_MOESM1_ESM.pdf]

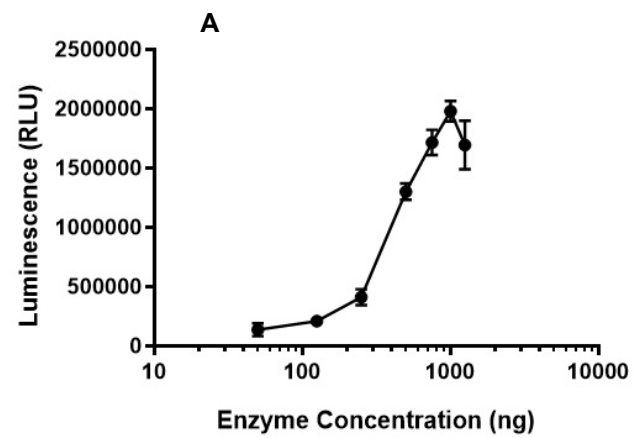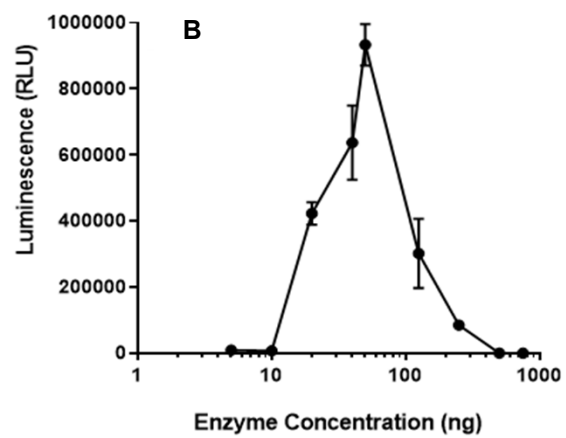

Supplement: Supplementary file 2 — Additional file 2: Figure S2. Bioluminescence assay with different amount of W-Enzyme and hydrolyzed sugar. A With UDP-Glo assay increasing enzyme amount is correlated to increasing activity (maximum with 1000 ng). B In CMP-Glo assay maximum activity was observed with 50 ng of enzyme. Data point indicates the mean and error bars represent standard deviation. Each panel illustrates representative examples of three individual experiments. All experiments were run with two replicates. [file 13104_2021_5831_MOESM2_ESM.pdf]

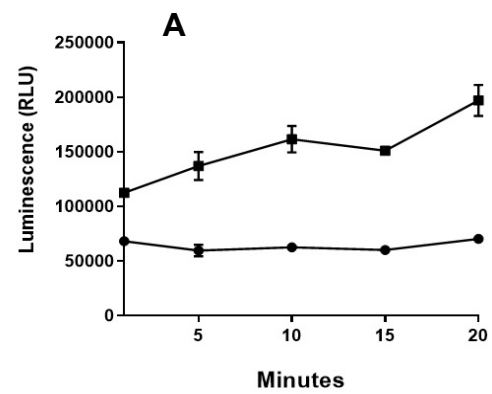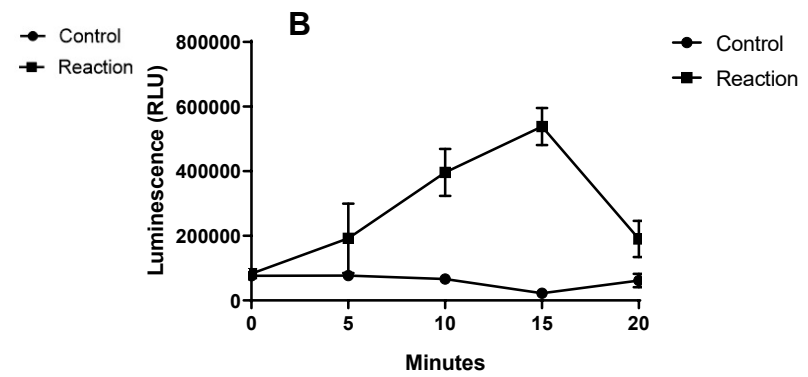

Supplement: Supplementary file 3 — Additional file 3: Figure S3. Time course reactions using hydrolyzed sugar acceptor. A CMP-Glo results: The plot of results linearity over 10 minutes whereas for B UDP-Glo results: this linearity was obtained over 15 minutes. All reactions run in duplicate. Data point indicates the mean and error bars represent standard deviation. Each panel illustrates representative examples of four individual experiments. All experiments were run with two replicates. [file 13104_2021_5831_MOESM3_ESM.pdf]

A

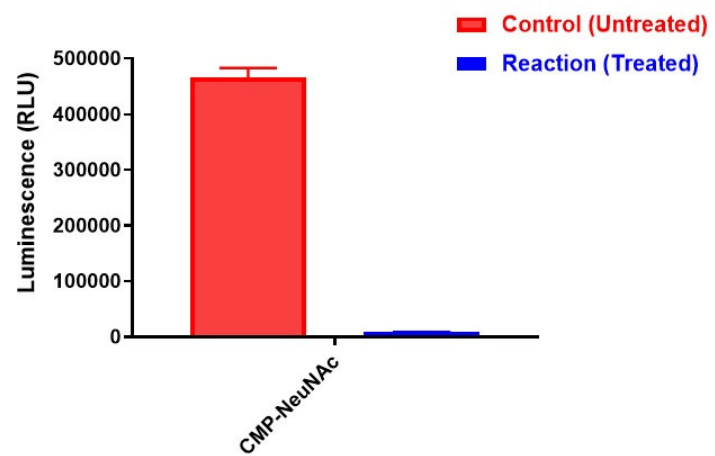

B

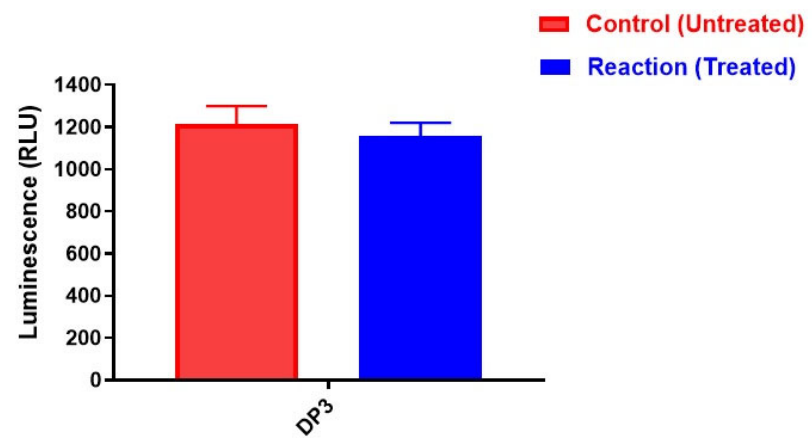

Supplement: Supplementary file 4 — Additional file 4: Figure S4. Alkaline phosphatase treatment of NmW substrates. A CMP-NeuNAc solutions were pre-treated with alkaline phosphatase. B DP3 trimer was subjected to phosphatase treatment. Each panel illustrates representative examples of three individual experiments. All experiments were run with three replicates. [file 13104_2021_5831_MOESM4_ESM.pdf]

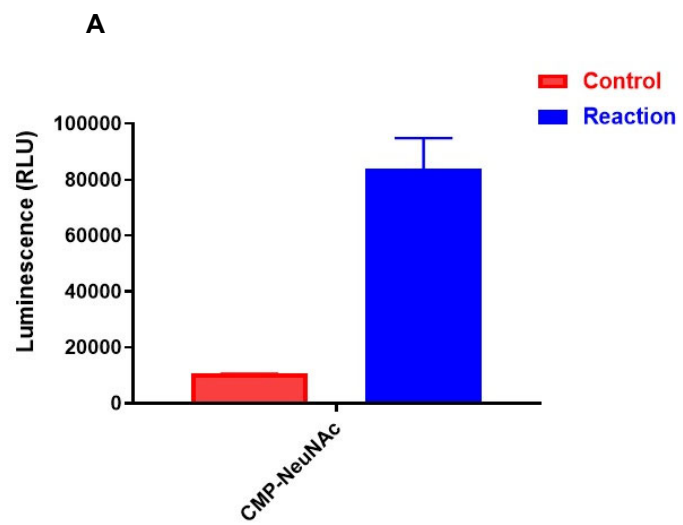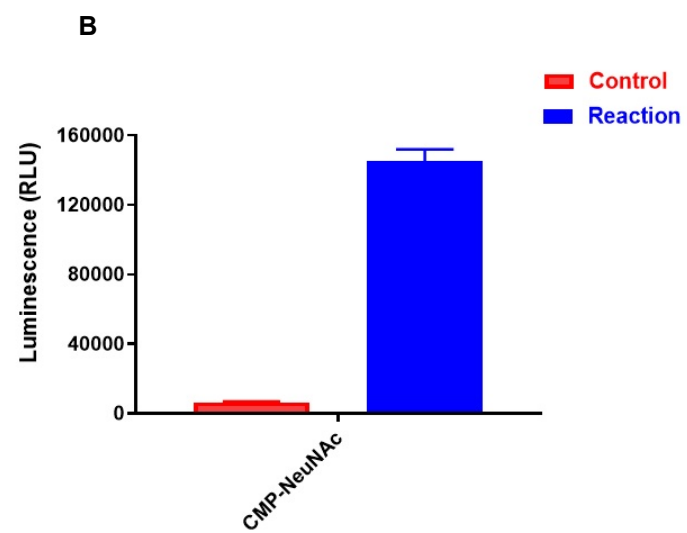

Supplement: Supplementary file 6 — Additional file 6: Figure S5. NmW capsule polymerase reaction using A untreated CMP-NeuNAc and B AP-treated CMP-NeuNAc in the UDP-Glo assay. The background signal with untreated CMP-NeuNAc was higher compared to treated. Higher overall luminescence signal was observed with treatment. Each panel illustrates representative examples of three individual experiments. All experiments were run with three replicates. [file 13104_2021_5831_MOESM6_ESM.pdf]

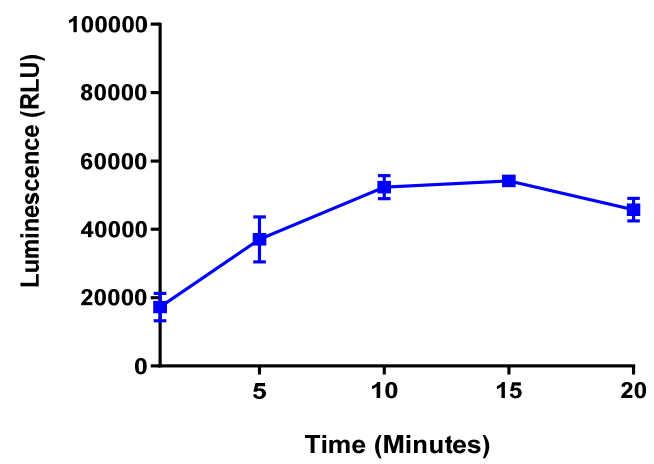

Supplement: Supplementary file 7 — Additional file 7: Figure S6. Time course experiment using DP3 acceptor in the UDP-Glo assay. The enzymatic reaction was found to be linear over 10 minutes. Data point indicates the mean and error bars represent standard deviation. This panel illustrates representative examples of three individual experiments. All experiments were run with three replicates. [file 13104_2021_5831_MOESM7_ESM.pdf]
