# Supplementary material for: Investigation of bioluminescence-based assays for determination of kinetic parameters for the bifunctional Neisseria meningitidis serogroup W capsule polymerase
Source: BMC Res Notes. 2021 Nov 18;14:417. doi: 10.1186/s13104-021-05831-1 (PMC8600345; doi:10.1186/s13104-021-05831-1)
Supplement: Supplementary file 5 — Additional file 5: Supplemental Methods. [file 13104_2021_5831_MOESM5_ESM.docx]

**SUPPLEMENTAL METHODS**

*Alkaline Phosphatase Treatment of Nucleotide Donor Sugars CMP-Neu5Ac and DP3* acceptor were treated using Alkaline Phosphatase (AP) Enzyme (20U/µl). A reaction was run by adding 10 units of AP (0.5 µL) to CMP-NeuNAc (1 or 10 mM) and DP3 (1 mM) respectively in 50 mM Tris, 1 mM MgCl_2_ buffer (pH 9.0). Reactions were incubated at 37ºC for 10 min. Treated material was separated from alkaline phosphatase using an Amicon Ultra 10K centrifugal filter at 7500 RPM. Treated nucleotide donor sugar was collected in the filtrate.
